# Aldose Reductase Differential Inhibitors in Green Tea

**DOI:** 10.3390/biom10071003

**Published:** 2020-07-06

**Authors:** Francesco Balestri, Giulio Poli, Carlotta Pineschi, Roberta Moschini, Mario Cappiello, Umberto Mura, Tiziano Tuccinardi, Antonella Del Corso

**Affiliations:** 1 Department of Biology, University of Pisa, Biochemistry Unit, via S. Zeno, 51, 56123 Pisa, Italy; francesco.balestri@unipi.it (F.B.); carlotta.pineschi@gmail.com (C.P.); roberta.moschini@unipi.it (R.M.); mario.cappiello@unipi.it (M.C.); umberto.mura@unipi.it (U.M.); 2 Interdepartmental Research Center Nutrafood “Nutraceuticals and Food for Health”, University of Pisa, 56124 Pisa, Italy; 3 Department of Pharmacy, University of Pisa, via Bonanno 6, 56126 Pisa, Italy; giulio.poli@unipi.it (G.P.); tiziano.tuccinardi@unipi.it (T.T.)

**Keywords:** epigallocatechin gallate, gallic acid, green tea, aldose reductase, differential inhibitors, ARDIs, diabetic complications

## Abstract

Aldose reductase (AKR1B1), the first enzyme in the polyol pathway, is likely involved in the onset of diabetic complications. Differential inhibition of AKR1B1 has been proposed to counteract the damaging effects linked to the activity of the enzyme while preserving its detoxifying ability. Here, we show that epigallocatechin gallate (EGCG), one of the most representative catechins present in green tea, acts as a differential inhibitor of human recombinant AKR1B1. A kinetic analysis of EGCG, and of its components, gallic acid (GA) and epigallocatechin (EGC) as inhibitors of the reduction of L-idose, 4-hydroxy2,3-nonenal (HNE), and 3-glutathionyl l-4-dihydroxynonanal (GSHNE) revealed for the compounds a different model of inhibition toward the different substrates. While EGCG preferentially inhibited L-idose and GSHNE reduction with respect to HNE, gallic acid, which was still active in inhibiting the reduction of the sugar, was less active in inhibiting HNE and GSHNE reduction. EGC was found to be less efficient as an inhibitor of AKR1B1 and devoid of any differential inhibitory action. A computational study defined different interactive modes for the three substrates on the AKR1B1 active site and suggested a rationale for the observed differential inhibition. A chromatographic fractionation of an alcoholic green tea extract revealed that, besides EGCG and GA, other components may exhibit the differential inhibition of AKR1B1.

## 1. Introduction

Drinking green tea is common both in the east and the west. The beneficial properties associated with this beverage are widely recognized [1,2,3]. Green tea is referred to for its general antioxidant/detoxifying and anticancer activities, although some concerns regarding its abuse have been raised [4]. Epigallocatechin gallate (EGCG), the ester of epigallocatechin (EGC) and gallic acid (GA), is certainly the most abundant catechin and perhaps the key component in the green tea [5]. The effectiveness of this polyphenolic compound in a variety of cell pathological/stress conditions is associated with its antioxidant/inflammatory [6,7,8,9] and anticarcinogenic [10,11,12,13] action. Moreover, EGCG has also been reported as a liver and kidney protector against different stimuli [5,14,15,16] and appears to intervene in reducing apoptotic phenomena above all induced by hyperglycemic conditions [17,18,19].

There is also evidence of the possible effects of the two structural components of EGCG. In fact, both EGC and GA have been reported to possess anticarcinogenic effects [20,21,22,23,24] and GA displays both antioxidant and prooxidant features, depending on the cell types [25,26,27].

Aldose reductase (EC 1.1.1.21; AKR1B1), the first enzyme in the polyol pathway, is likely implicated in a variety of pathological states such as nephropathies, retinopathies, neuropathies, cardiopathies, cataract, and cancer, mainly related to its action on glucose in hyperglycaemic conditions [28,29,30,31,32,33,34]. It is worth noting that this enzyme has a detoxifying function, linked to the ability of reducing 4-hydroxy-*trans*-2-nonenal (HNE), one of the preeminent products of lipid peroxidation [35]. AKR1B1 also has pro-inflammatory action, since it converts the glutathionyl-HNE adduct (GSHNE) into 3-glutathionyl-1,4-dihydroxynonane (GSDHN) [36,37], which in turn activates the NF-κB signaling cascade [38,39,40]. All this has increased the interest in AKR1B1 as a target for inhibition [30,41].

The lack of a parallel between in vitro AKR1B1 inhibitors (ARIs) discovery and the development of ARI-based drugs, together with the assessment of particular features of the enzyme, led to the proposal of a novel approach that controls the AKR1B1 activity through an intra-site differential inhibition [42]. Aldose reductase differential inhibitors (ARDIs) act differentially on the AKR1B1 activity depending on the nature of the substrate the enzyme is working on [43]. In this respect, a useful ARDI would favour the inhibition of the sugar and/or GSHNE reduction, leaving unaffected (or at least less affected) the detoxifying action of AKR1B1 in reducing HNE and other alkanals and alkenals. For some vegetable extracts there is a differential inhibitory action between L-idose and HNE [44]. In addition, soyasaponins appear to contribute as ARDIs to the AKR1B1 inhibitory capacity of Zolfino bean extracts [45,46].

In this paper, we provide evidence that EGCG and GA, which have been reported to act as conventional ARIs [47,48,49,50] may also act as ARDIs by differentially acting on the reduction of different substrates. The effects exerted of gallic acid (GA) and epigallocatechin (EGC) are compared. A computational study approach performed on the enzymatic systems defined different interaction modes for the three substrates with the enzyme active site and suggested a rationale for the differential inhibition observed. These results, besides extending the known multi-target activity of EGCG and GA, suggest new beneficial actions of green tea, which could favourably intervene in the onset of diabetic complications.

## 2. Materials and Methods

### 2.1. Materials

EGCG, GA, EGC, NADPH, and L-idose were obtained from Carbosynth (Compton, England). Bovine serum albumin (BSA), D,L-dithiothreitol (DTT), D,L-glyceraldehyde (GAL), EDTA, ultrapure acetone (99.9% purity), and ultrapure methanol (99.9% purity) were purchased from Sigma-Aldrich (Saint Louis, MO, USA); YM10 ultrafiltration membranes were obtained from Merck-Millipore (Darmstadt, Germany); Bondelut C18, solid phase extraction columns, octadecyl sorbent, 60 Å pore size, surface area 500 m^2^/g (5 g resin), column volume 25 mL, were obtained from UCT Inc. (Bristol, PA, USA). All other chemicals were of a reagent grade. The green tea (*Camelia sinensis* L., leaf) freeze-dried extract was supplied by Aboca S.p.A., and prepared as follows; leaves of green tea were extracted (weight ratio of leaves to solvent 1:8) with ethanol 70% (ethanol:water 70:30, *v*/*v*). The herb was loaded in the extractor equipped with a perforated basket and the extraction solvent was added. The extraction process is characterized by the solvent recirculation from the bottom to the top using a mechanical pump, while the herb is standing in the basket. After 8–10 h, at 40 °C, the alcoholic herb’s mixture was dropped for 1 h and the resulting extract was filtered on paper by means of a sheet filtration system to remove all the exhausted herb small pieces. The resulting clarified extract was concentrated by ethanol evaporation using a vacuum concentration thin film plant. On reaching the concentration level 7:1 the concentration was interrupted and drying was completed by freeze-drying for 72 h. The resulting freeze-dried extract (ratio of weight dry plant material to final extract weight 3:1) was stored until use, away from light and moisture.

### 2.2. Assay of Aldose Reductase

The AKR1B1 activity was determined at 37 °C, as previously described [51], monitoring the decrease in absorbance at 340 nm linked to NADPH oxidation (ε_340_ = 6.22 mM^−1^.cm^−1^) using a Biochrom Libra S60 spectrophotometer. The standard assay mixture (0.7 mL) contained in a 0.25 M sodium phosphate buffer pH 6.8, 0.18 mM NADPH, 0.4 M ammonium sulfate, 0.5 mM EDTA, and 4.7 mM GAL. One unit of enzyme activity is the amount of enzyme that catalyzes the conversion of 1 µmol of substrate/min in the above assay conditions. The same assay conditions were adopted in the inhibition studies with L-idose, HNE, or GSHNE as substrates.

IC_50_ values were determined by the non-linear regression analysis using GraphPad Prism version 6.0 (GraphPad Software, San Diego, CA, USA); for each value the 95% confidence limits (CLs) are reported.

### 2.3. Purification of Human Recombinant AKR1B1

The human recombinant AKR1B1 was expressed and purified to electrophoretic homogeneity, as previously described [52]. The specific activity of the purified enzyme was 5.3 U/mg. The purified enzyme was stored at −80 °C in a 10 mM sodium phosphate buffer pH 7.0 containing 2 mM DTT and 30% (*w*/*v*) glycerol. Before use, AKR1B1 was extensively dialyzed against 10 mM of a sodium phosphate buffer pH 7.0.

### 2.4. Preparation of a Green Tea “Water-Soluble Fraction”

The freeze-dried green tea extract (see Materials) was suspended in ultrapure water (50 mg in 1.6 mL) and centrifuged for 30 min at 13,000× *g* (Beckman Microfuge 18 Centrifuge) at room temperature. The sediment was washed twice by the addition of 200 µL of water, followed by centrifugation as above for 10 min. The three supernatants were then collected, thus generating the “water-soluble fraction”. In order to determine the relative content of the water-insoluble fraction, the sediment was washed twice by the addition of 200 µL of water, followed by centrifugation as above for 10 min. The residual water on the pellet was removed by freeze/vacuum rotary evaporation followed by a drying step at 60 °C for 1 h. The actual concentration (mg/mL) of the water-soluble fraction was determined by subtracting the weight of the dried water insoluble pellet from the weight of the starting material.

### 2.5. Green Tea Extracts Fractionation

Before use, Bondelut C18 columns were conditioned, which entailed a 25 mL free flowing of ultrapure acetone followed by a 25 mL free flowing of ultrapure methanol. The column was then washed at a flow rate of 30 mL/h for 5 h with methanol, and then overnight at 4 °C with ultrapure water. The column was loaded with 16 mg of water-soluble tea extract. The elution steps were performed at a flow of 30 mL/h and 2 mL fractions were collected. Elution was monitored by measuring the absorbance of the eluted fractions at 254 nm. The inhibitory effectiveness of eluted fractions was tested on L-idose and HNE reduction. In order to prevent the possible interference of methanol in the determination of enzyme activity, the alcohol concentration in the assay was kept lower than 3% (*v*/*v*). Individual fractions were dried, suspended in one tenth of their original volumes in their respective water/methanol eluent mixtures, and then used (20 µL) to assay their inhibitory capability. Commercial EGCG, GA, and EGC were analyzed individually as above, applying 5 µmoles of each compound to the column in the above described conditions.

### 2.6. Kinetic Analysis

The kinetic analysis of the experimental rate measurements with different substrates (S) in the presence of inhibitors (I) was performed on the classical inhibition model of action. Here, (I) is considered to intervene into a steady state condition of the reaction, being free to interact with the free enzyme (E), the ES complex or with both, through a rapid equilibrium. Data were analyzed by Hanes-Woolf plots [53]. The apparent dissociation constants ^*app*^*K_i_’* (for the ESI complex) and *^app^K_i_* (for the EI complex) were determined from secondary plots of 1/*^app^V_max_* and *^app^K_M_*/*^app^V_max_* as a function of the inhibitor concentration, respectively.

### 2.7. Molecular Docking

The X-ray structure of human AKR1B1 in complex with D-glyceraldehyde and the NADP cofactor (PDB code 3V36) [54], as well as the co-crystal structure of human AKR1B1 in complex with a nitrofuryl-oxadiazol inhibitor (PDB code 2IKH) [55], were downloaded from the Protein Data Bank [56]. Molecular docking calculations were performed with GOLD 5.1 (CCDC Software Ltd., Cambridge, UK) [57] using the piecewise linear potential (PLP) fitness function. The region of interest for the docking calculations included all the residues which stayed within 10 Å from the bound ligand in the corresponding X-ray structures. The ligands were subjected to 100 genetic algorithm runs, in which the “allow early termination” option was deactivated, while the possibility for the ligand to flip ring corners was activated. All other settings were left as their defaults. The default root-mean-squared deviation (RMSD) threshold for pose clustering was set to 2.0 Å for GSHNE, EGC, and EGCG, while for D-glucose, L-idose, HNE, and GA, which are low MW ligands, a cut-off of 1.5 Å was used. The best docked conformation of each cluster of solution was considered for each ligand in each docking study.

### 2.8. Molecular Dynamics Simulations

Molecular dynamics (MD) simulations were performed with AMBER 16 [58] using the ff14SB force field. General amber force field (GAFF) parameters were used for the ligands, the substrates, and the cofactor, whose partial charges were calculated with the AM1-BCC method as implemented in the Antechamber suite of AMBER 16 (University of California, San Francisco, CA, USA). All simulations were performed using particle mesh Ewald electrostatics, a cut-off of 10 Å for non-bonded interactions and periodic boundary conditions. All bonds involving hydrogens were kept rigid using the SHAKE algorithm and a simulation step of 2.0 fs was thus employed. Each analyzed complex was placed in a rectangular parallelepiped water box, using the TIP3P explicit solvent model for water, and solvated with a 15 Å water cap. Sodium ions were added as counterions for the neutralization of the systems. Initially, the systems were energy minimized through two minimization steps, including 5000 cycles of steepest descent followed by the conjugate gradient, until a convergence of 0.05 kcal/Å mol. In the first step, the protein was maintained rigid with a position restraint of 100 kcal/mol·Å^2^, thus minimizing only the positions of the water molecules. In the second step, the whole system was energy minimized by applying a harmonic potential of 10 kcal/mol·Å^2^ only to the protein α carbons. The minimized complexes were then used as the starting point for the MD simulations, applying a protocol adapted from previous studies [59,60]. A 0.5 ns constant-volume simulation, in which the temperature of the system was raised from 0 to 300 K, was initially performed. The system was then equilibrated with 3 ns of constant-pressure simulation, keeping a constant temperature of 300 K with the use of Langevin thermostat. Finally, 46.5 ns of MD simulations with constant pressure and temperature conditions were performed, for a total of 50 ns of simulation. In all MD steps, the harmonic potential of 10 kcal/mol·Å^2^ on the protein α carbons was maintained.

### 2.9. Binding Energy Evaluation

Relative binding free energy evaluations were performed using AMBER 14 (University of California, San Francisco, CA, USA), as previously performed [61,62]. The trajectories extracted from the last 30 ns of each simulation were used for the calculation, for a total of 300 snapshots (at time intervals of 100 ps). Van der Waals, electrostatic and internal interactions were calculated with the SANDER module of AMBER 14, whereas the Generalized Born and the Poisson−Boltzman methods were employed to estimate polar energies through the MM-PBSA module of AMBER 14. Gas and water phases were represented using dielectric constants of 1 and 80, respectively, while nonpolar energies were calculated with the MOLSURF program. The entropic term was considered as approximately constant in the comparison of the ligand-protein energetic interactions.

### 2.10. Other Methods

The protein concentration was determined by the Coomassie blue staining method [63], using BSA as a standard protein. HNE was synthetized as previously described [64]. GSHNE was prepared as previously described [65] by incubating GSH and HNE (1.5:1 molar ratio) and monitoring the time course of GSH consumption [66]. The statistical analysis was performed using a one-way ANOVA test (Tukey’s multiple comparison test) carried out with Graphpad 6.0 (GraphPad Software, San Diego, CA, USA).

## 3. Results and Discussion

### 3.1. Evidence of Differential Inhibition for AKR1B1-Dependent Reactions

**Figure 1:**
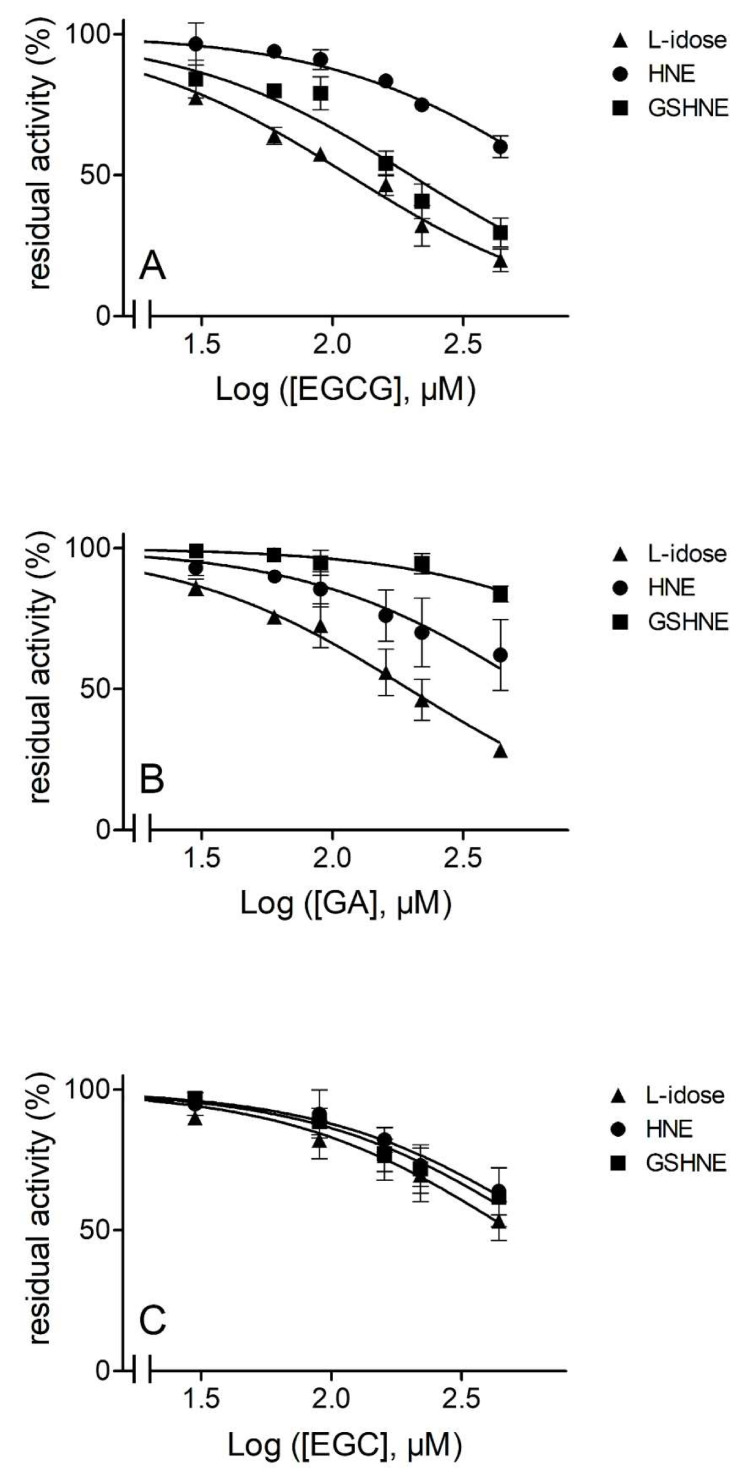
Inhibition curves of aldose reductase (AKR1B1) by epigallocatechin gallate (EGCG), gallic acid (GA), and epigallocatechin (EGC). Commercial standards of EGCG *Panel* (**A**), GA *Panel*, (**B**) and EGC *Panel* (**C**) were used to evaluate the dose-dependent inhibitory effect on 8 mU of AKR1B1 acting on 8 mM l-idose (triangles), 0.03 mM 4-hydroxy-2-nonenal (HNE, circles), and 0.07 mM 3-glutathionyl-4-hydroxynonanal (GSHNE, squares). Error bars (when not visible are within the symbol size) represent the standard deviations of the mean from three to five independent measurements.

The ability of EGCG and its basic components, GA and EGC to act as ARDIs towards L-idose and GSHNE with respect to HNE, is shown in Figure 1, which refers to assays conducted with the concentrations of sugar substrate at the millimolar level and GSHNE and HNE at the micromolar level. Both L-idose, used as a sugar substrate mimicking D-glucose, and GSHNE, the pro-inflammatory AR substrate, were inhibited by EGCG with comparable IC_50_ values of 115 µM (101–132, 95% CLs) and 199 µM (148–268, 95% CLs), respectively.

At these inhibitor concentrations, the residual activity measured for HNE reduction (IC_50_ 709 µM, 622–808, 95% CLs) was still 80–90% of the activity value measured in the absence of EGCG (Figure 1A). Thus, in these conditions, a comparable significant (*p* < 0.0001) ARDI action for the reduction of both L-idose and GSHNE with respect to HNE was exerted by EGCG.

Looking at the inhibitory action of GA (Figure 1B), the IC_50_ value measured for L-idose reduction was 196 µM (175–219, 95% CLs). At this inhibitor concentration, the residual activity measured for HNE reduction (IC_50_ 586 µM, 459–748, 95% CLs) was approximately 75% of the control activity measured in the absence of the inhibitor. On the other hand, GA was a less potent inhibitor on GSHNE (IC_50_ 2.5 mM, 1.3–4.8, 95% CLs) with respect to findings observed for the other two substrates. This makes GA still able to act as an ARDI, although the potential to act against inflammatory events linked to GSHNE reduction is significantly attenuated. Finally, as shown in Figure 1C, EGC appeared to be poorly effective as ARI, and completely ineffective as ARDI, as the inhibitory action was essentially unaffected by the nature of the substrate, with IC_50_ values ranging from 500 to 700 µM. These results suggest the essentially passive role of the catechin moiety of EGCG in terms of inhibition, but a significant effect of GA in the assembly of an active ARDI scaffold.

### 3.2. Inhibition Kinetic Analysis

**Figure 2:**
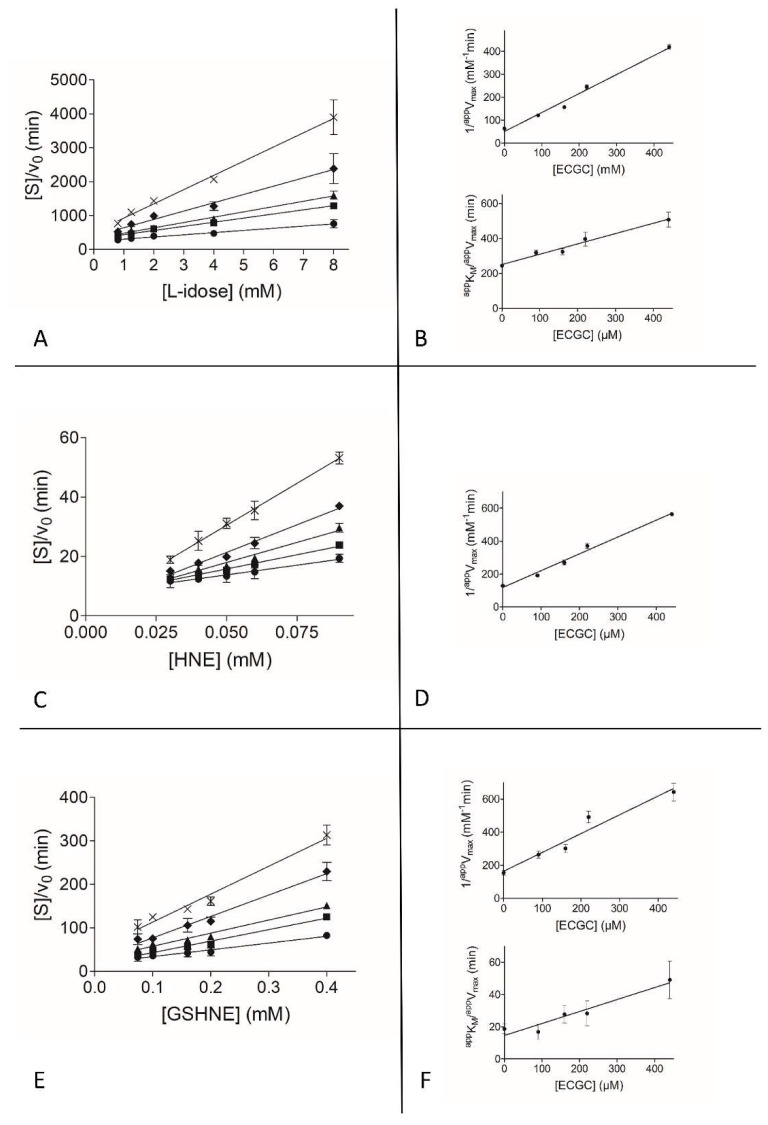
Kinetic characterization of EGCG as an AKR1B1 inhibitor. *Panel* (**A**), *Panel* (**C**), and *Panel* (**E**) are the Hanes-Woolf plots obtained when the activity of the purified enzyme (8 mU) was measured at the indicated concentrations of L-idose, HNE, and GSHNE as substrates, in the absence (●) or in the presence of the following inhibitor concentrations: (■) 90 µM, (▲) 160 µM, (♦) 220 µM, (×) 440 µM. *Panel* (**B**), *Panel* (**D**), and *Panel* (**F**) refer to the secondary plots of the slopes (1/*^app^V_max_*) and the ordinate intercept (*^app^K_M_*/*^app^V_max_*) of the relative primary plot, as a function of the inhibitor concentration. *Panel* (**A**) and *Panel* (**B**) refer to l-idose; *Panel* (**C**) and *Panel* (**D**) refer to HNE; *Panel* (**E**) and *Panel* (**F**) refer to GSHNE. Error bars (when not visible are within the symbol size) represent the standard deviations of the mean from at least three independent measurements.

**Figure 3:**
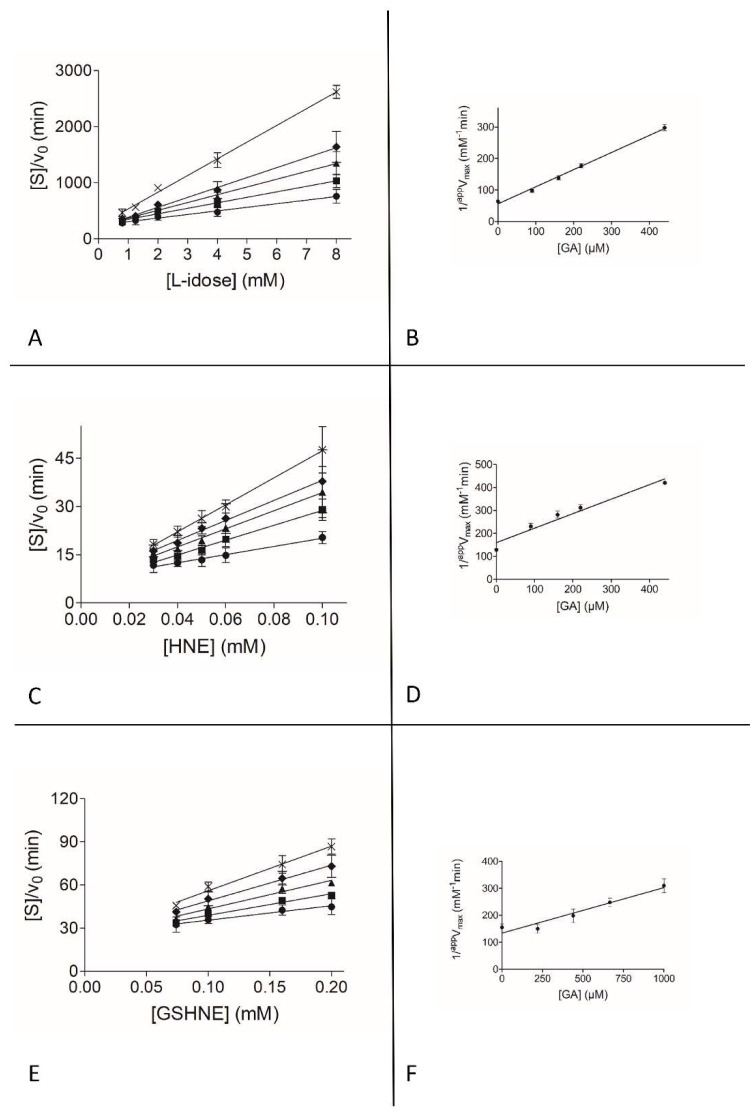
Kinetic characterization of GA as an AKR1B1 inhibitor. *Panel* (**A**), *Panel* (**C**), and *Panel* (**E**) are the Hanes-Woolf plots obtained when the activity of the purified enzyme (8 mU) was measured at the indicated concentrations of L-idose and HNE as substrates, in the absence (●) or in the presence of the following inhibitor concentrations: (■) 90 µM, (▲) 160 µM, (♦) 220 µM, (×) 440 µM. When GSHNE was used as a substrate, symbols refer to the following inhibitor concentrations: (●) zero, (■) 220 µM, (▲) 440 µM, (♦) 670 µM, (×) 1000 µM. *Panel* (**B**), *Panel* (**D**), and *Panel* (**F**) refer to the secondary plots of the slopes (1/*^app^V_max_*) and the ordinate intercept (*^app^K_M_*/*^app^V_max_*) of the relative primary plot, as a function of the inhibitor concentration. *Panel* (**A**) and *Panel* (**B**) refer to l-idose; *Panel* (**C**) and *Panel* (**D**) refer to HNE; *Panel* (**E**) and *Panel* (**F**) refer to GSHNE. Error bars (when not visible are within the symbol size) represent the standard deviations of the mean from at least three independent measurements.

**Figure 4:**
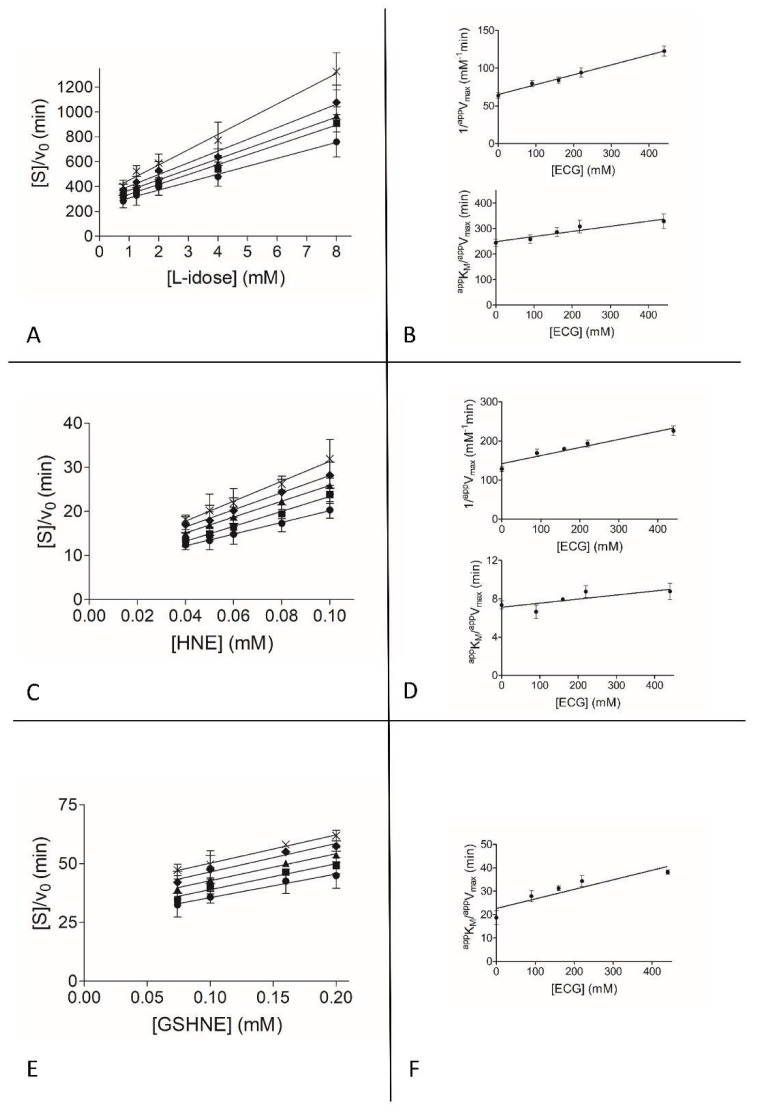
Kinetic characterization of EGC as an AKR1B1 inhibitor. *Panel* (**A**), *Panel* (**C**), and *Panel* (**E**) are the Hanes-Woolf plots obtained when the activity of the purified enzyme (8 mU) was measured at the indicated concentrations of L-idose, HNE, and GSHNE as substrates, in the absence (●) or in the presence of the following inhibitor concentrations: (■) 90 µM, (▲) 160 µM, (♦) 220 µM, (×) 440 µM. *Panel* (**B**), *Panel* (**D**), and *Panel* (**F**) refer to the secondary plots of the slopes (1/*^app^V_max_*) and the ordinate intercept (*^app^K_M_*/*^app^V_max_*) of the relative Hanes-Woolf plot, as a function of the inhibitor concentration. *Panel* (**A**) and *Panel* (**B**) refer to l-idose; *Panel* (**C**) and *Panel* (**D**) refer to HNE; *Panel* (**E**) and *Panel* (**F**) refer to GSHNE. Error bars (when not visible are within the symbol size) represent the standard deviations of the mean from at least three independent measurements.

To gain insights into the observed differential inhibition ability of EGCG and GA, and into the apparent ineffectiveness of EGC as ARDI, a detailed kinetic analysis on a classical inhibition model of action (see Methods) was performed with the three different substrates. Figure 2, Figure 3 and Figure 4 show the graphical analysis of the data obtained for the three different substrates.

The resulting apparent enzyme-substrate-inhibitor complex dissociation constant (*^app^K’_i_*) and the apparent enzyme-inhibitor complex dissociation constant (*^app^K_i_*) values are reported in Table 1.

When acting on the L-idose reduction, EGCG shows a mixed type of inhibition in which the targeting of the ES complex prevails over the targeting of the free enzyme, with a *K_i_* at least 7-fold higher than *K’_i_*. This preferential binding of EGCG to the ES complex appears to be strongly enhanced in the case of HNE reduction. In fact, in HNE reduction the inhibition occurs through a typical uncompetitive model of action. A completely different situation occurs for the inhibitory action of EGCG on the reduction of GSHNE. In this case, in fact, although with a slightly reduced efficiency (*K’_i_* approximately 1.2- to 2.4-fold higher than for the HNE and L-idose reduction, respectively), the inhibitor binds the free enzyme and the ES complex with essentially the same effectiveness (*K_i_*/*K’_i_* 1.3). Thus, for this substrate an apparently purely non-competitive type of inhibition occurs.

Looking at the absolute values of the apparent inhibition kinetic constants measured for different substrates, despite showing a preferential action on L-idose reduction, it is evident that EGCG cannot be defined as a complete differential inhibitor [67]. In fact, it also affects HNE reduction—although to a lesser extent. However, in conditions such as those shown in Figure 1, that mimic possible in vivo hyperglycemia, an appreciable differential inhibitory action is generated. This is true both to L-idose and GSHNE reduction compared to HNE reduction.

GA appears to exert a differential inhibition of L-idose with respect both to HNE and GSHNE (Figure 1B), and an uncompetitive type of inhibition is observed (Table 1). There is a parallel significant drop in inhibition efficiency, especially evident for GSHNE (*K’_i_* of GA approximately five times higher than *K’_i_* of EGCG).

Finally, looking at EGC, which inhibits the reaction irrespectively of the substrate used (Figure 1C), the kinetic analysis explains the rather poor ability of this molecule to inhibit AKR1B1.

The basic assumption on the concurrent action of different factors leading to differential inhibition, is thus that different substrates must approach and/or allocate into the enzyme active site differently. This is what the following computational analysis (see below) reveals for the L-idose, HNE, and GSHNE interacting at the active site of the AKR1B1.

### 3.3. Computational Study of AKR1B1 Differential Inhibition

The basic requirement for an inhibitor to exert an intra site differential inhibition stands on different interactions between different substrates and the enzyme [67]. Thus, in order to evaluate different binding modes of the substrates, an extensive computational study was performed, involving molecular docking, molecular dynamics (MD) simulations, and ligand-protein binding energy evaluations. In addition, despite that the inhibition results (i.e., *^app^K_i_’* *^app^K_i_* values) from the classical inhibition kinetic approach are not univocal, we attempted to correlate them with calculated interactive models.

#### 3.3.1. Substrates Allocation at the Enzyme Active Site

**Figure 5:**
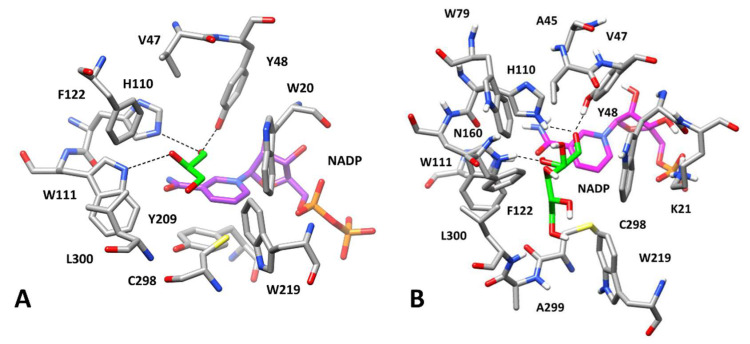
Interactions of AKR1B1 with aldose substrates. *Panel* (**A**): X-ray structure of AKR1B1 in complex with D-glyceraldehyde and NADP (PDB code 3V36). *Panel* (**B**): Minimized average structures of AKR1B1 in complex with D-glucose, as emerging from the computational analysis. Aldoses are shown in green while the portion of the cofactor adjacent to the ligand binding site is shown in magenta. Relevant protein residues (shown in grey) are indicated. For all components, oxygen atoms are in red, sulfur atoms in yellow, nitrogen atoms in blue, while phosphorous atoms of the cofactor are shown in orange.

We initially focused on predicting the potential binding mode of D-glucose, HNE and GSHNE, which are natural substrates of AKR1B1, as well as L-idose which was used for measuring the AKR1B1 activity in our experimental assays as an effective substrate alternative to glucose [52]. Initially, a robust docking study using the GOLD software [57] was performed to generate possible binding conformations for D-glucose and L-idose. The ligands were docked into the X-ray structure of a human AKR1B1 in complex with D-glyceraldehyde (D-GAL) and the NADP cofactor (PDB code 3V36). For each ligand, 100 docking solutions were generated and then clustered, based on their reciprocal RMSD, in order to identify different potential binding modes corresponding to the different clusters of solutions. In the reference crystallographic complex, the carbonyl group of D-GAL, is located in the so-called anion binding pocket, constituted by Y48, H110, W111, and the cofactor nicotinamide moiety, and interacts with the catalytic residues Y48 and H110 through two H-bonds (Figure 5A).

Thus, we assumed that all other AKR1B1 substrates will bind the AKR1B1 catalytic site by locating the carbonyl group within the anion binding pocket in a similar way. For this reason, the binding modes generated for D-glucose and L-idose in the AKR1B1 binding site were filtered, only considering those in which both H-bonds between Y48, H110, and the carbonyl oxygen of the substrates were observed. With this restriction, seven different poses for D-glucose and three for L-idose were selected. All were analyzed exploiting a 50 ns MD simulation protocol already used in several pose prediction studies [59,60]. Their reliability was successfully tested on the reference D-GAL-protein complex, in which both the binding disposition of D-GAL and its interactions with the protein residues were properly maintained.

With this approach, only two out of the seven possible D-glucose-AKR1B1 complexes predicted by docking were properly maintained throughout the whole simulation. In fact, only for complex 3 and complex 5 was the average RMSD of D-glucose disposition during the MD, with respect to the initial coordinates, found to be lower than 2.0 Å (1.6 and 1.8 Å, respectively). In fact, by visually inspecting the trajectories of the MD simulations, for the other remaining complexes (1, 2, 4, 6, and 7), the ligand tended to assume a similar disposition to that observed in either complex 3 (from complexes 4 and 6) or complex 5 (from complexes 1, 2, and 7).

In order to analyze the reliability of the predicted binding modes from a quantitative point of view, ligand-protein binding energy evaluations were performed in all seven ligand complexes using the MM-PBSA method (see Materials and Methods). The two most stable complexes showed a considerable binding energy difference, corresponding to about 5 kcal/mol, which suggested a high reliability for complex 3 (ΔPBSA = −19.2 kcal/mol) with respect to complex 5 (ΔPBSA = −15.5 kcal/mol). All other complexes showed intermediate binding energy values.

Interestingly, for complexes 4 and 6, in which the D-glucose binding pose actually converged into that of complex 3 after about 20 ns of MD, a higher binding energy (−18.3 and −18.5 kcal/mol, respectively) was obtained compared with that of complexes 1, 2, and 7 (ranging from −16.0 to −16.9 kcal/mol), in which D-glucose tended to assume a similar binding mode to that of complex 5. Based on these results, complex 3, shown in Figure 5B, was found to be the most reliable, from both a qualitative and a quantitative point of view. Here, the carbonyl group of D-glucose formed stable H-bonds with the catalytic residues H110 and Y48, which were maintained for most of the MD simulation. In addition, the H-bond with W111 detected for D-GAL in the reference complex was also observed. D-glucose formed another stable H-bond interaction with the carbonyl of the carboxamide group of the cofactor, which further contributes to anchoring the ligand to the anion binding pocket.

Finally, a transient and less stable H-bond between L300 and the C6-hydroxyl group of glucose was observed in the simulation. This H-bond network established among the substrate, cofactor, and proximal residues provides a possible explanation for the selectivity of AKR1B1 toward D-glucose over L-glucose, which is a very poor AKR1B1 substrate [51]. In fact, L-glucose, with the configuration of all the four chiral centers inverted, did not form the same pattern of interactions and could not assume the same disposition of D-glucose. In contrast, our computational protocol suggested a very similar binding pose for L-idose.

**Figure 6:**
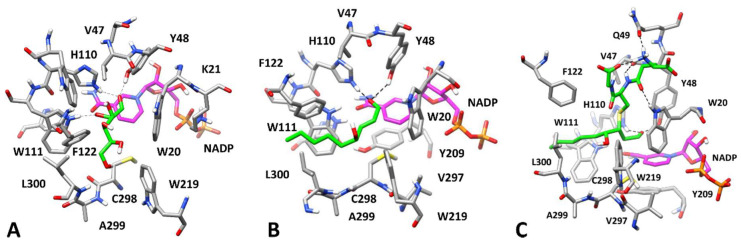
Minimized average structures of AKR1B1 in complexes with different substrates. *Panel* (**A**): L-idose; *Panel* (**B**): HNE; *Panel* (**C**): GSHNE. Substrates are shown in green. The portion of the cofactor adjacent to the ligand binding site is shown in magenta. Relevant protein residues (shown in grey) are indicated. For all components, oxygen atoms are in red, sulfur atoms in yellow, nitrogen atoms in blue, while phosphorous atoms of the cofactor are shown in orange.

In fact, regarding L-idose, while the MD analysis of the three poses selected by docking (complexes 1–3) highlighted their good stability (with average ligand RMSD between 1.5 and 1.9 Å), the energetic evaluations suggested that complex 1 was the most reliable one, with a ligand-protein interaction energy (ΔPBSA = −17.4 kcal/mol) of about 4 kcal/mol higher than that associated with the other complexes (−13.0 and −13.5 kcal/mol for complexes 2 and 3, respectively). As shown in Figure 6A, the binding mode predicted for L-idose is comparable with that of D-glucose, allowing the same interaction pattern, because of the strong structural analogy of the two epimers. These results highlight that the change in configuration at the C5 carbon atom of the two molecules does not affect their binding mode, which justifies their equivalence as AKR1B1 substrates [52].

The same docking/MD protocol adopted for aldoses was applied to predict the potential binding mode of HNE into the AKR1B1 binding site. However, in this case the unique cluster of poses generated by docking, preserving the proper substrate-protein interactions with the catalytic residues, was found to be highly unstable during the MD simulations. In fact, the ligand moved 5.3 Å away, on average, from its initial docking pose, with the hydrophobic tail fluctuating in the solvent-exposed region of the binding site.

Several ARIs appear to interact with AKR1B1 through their hydrophobic moieties upon inducing the rotation of L300 toward W219, which opens a mainly hydrophobic cavity (i.e., the specificity pocket) adjacent to the anion binding pocket [68]. Based on these considerations, and taking into account the hydrophobic features of HNE, we hypothesized that this substrate might interact with the enzyme by inducing the opening of the specificity pocket and thus placing its long hydrophobic tail inside this pocket. Thus, we docked HNE into the X-ray structure of a human AKR1B1 in complex with a nitrofuryl-oxadiazol inhibitor (PDB code 2IKH) that occupies the specificity pocket. Again, only a single binding mode preserving the substrate-protein interactions of the carbonyl with the catalytic residues was obtained and then analyzed with MD simulations. In this case, the substrate perfectly maintained its binding conformation, with an average RMSD of its disposition during the MD of about 1.2 Å, thus suggesting the high reliability for this enzyme-substrate complex.

As shown in Figure 6B, HNE forms the two expected H-bonds with the catalytic residues Y48 and H110, placing the lipophilic chain inside the specificity pocket mainly delimited by L300, W110, and F112, in which hydrophobic interactions with these residues strongly stabilize the ligand binding. No substantial interactions were detected for the hydroxyl group of HNE during the MD. In fact, the presence of the hydroxyl group at C4 does not appear to be critical for the enzyme activity as shown for *trans*-2-nonenal, which is a very good substrate for AKR1B1 with a specificity constant that is twice as high as that measured for HNE [44].

The binding energy evaluations estimated a ligand-protein interaction energy of −18.6 kcal/mol for this AKR1B1-HNE complex. In contrast, the binding free energy calculated for the other complex, in which the HNE tail is exposed to the solvent, was found to be 4 kcal/mol lower. This supports the hypothesis that the interaction of HNE occurs upon the opening of the specificity pocket. These results may help in explaining how a wide range of structurally different substrates, such as long-chain lipophilic aldehydes and smaller, highly hydrophilic aldoses, are all endowed with a high affinity for AKR1B1 [44,52].

On the basis of the binding mode predicted for HNE, we evaluated the potential binding disposition of GSHNE, assuming that this substrate would also interact with the same enzyme conformation. For this reason, GSHNE was docked into the same X-ray structure used for HNE. GSHNE exists as four different diastereoisomers that behave differently as AKR1B1 substrates. In particular, the diastereoisomer couple 3R/S,4R-GSHNE displays more favorable kinetic parameters with respect to the diastereoisomer couple 3R/S,4S-GSHNE with an approximately 20 times higher specificity constant [69].

3S,4R-GSHNE and 3R,4R-GSHNE were then subjected to docking calculations, MD simulations, and binding free energy evaluations. None of the 10 putative binding modes selected by docking of 3S,4R-GSHNE, displaying the key H-bonds interactions with Y48 and H110, were shown to generate reliable AKR1B1-GSHNE complexes. As reported in Table S1, the top-scored complex in terms of the ligand-protein binding affinity (complex 9) presented a binding energy value which was close to the one estimated for other complexes, and did not show a particular stability, with a higher average RMSD of ligand disposition than that observed for other complexes.

The analysis performed with 3R,4R-GSHNE produced different results [69]. In this case, one of the 12 suitable complexes generated by docking and analyzed through MD simulations (complex 3) was found to outperform all the others from both a qualitative and quantitative point of view, showing a binding energy of −30.0 kcal/mol and an average RMSD of ligand disposition of 1.9 Å (Table S2).

The binding mode corresponding to complex 3 was thus identified as the most reliable and was used in further studies (Figure 6C). In this complex, the HNE moiety of the adduct assumes a comparable disposition to that observed for the free aldehyde, with the carbonyl group within the anion binding pocket, stably interacting through two H-bonds with Y48 and H110, and with the lipophilic tail placed inside the specificity pocket. The glutathionyl moiety of the molecule interacts with residues located in the upper part of the binding site. The ligand is well anchored to the enzyme thanks to its central amide group, which forms two H-bonds with the backbone oxygen of V47 and the indole moiety of W20; a third H-bond is formed between the positively charged amino group of GSHNE and the side chain of Q49. Finally, there is an intramolecular H-bond between the same group and the glycine-derived carbonyl moiety, which helps to stabilize the binding conformation of the ligand.

#### 3.3.2. Inhibition by EGCG and Derivatives in the Reduction of Different Substrates

Once reliable binding modes had been identified for D-glucose and for the three different substrates used in the present kinetic study of AKR1B1, we attempted to clarify the potential mechanism behind the differential inhibitory activity of GA and EGCG. In our inhibition model, the kinetic analysis shows that GA preferentially inhibits the reduction of L-idose over HNE and GSHNE, showing for all the substrates an apparent uncompetitive model of action, essentially targeting the ES complexes (Table 1). On this basis, GA was docked into the previously generated structure of AKR1B1 in complex with L-idose (Figure 6A) considering the substrate as part of the structure; the aim was to predict the occurrence of a potential enzyme-substrate-inhibitor (ESI) complex. All ten resulting binding modes produced by docking were considered and analyzed with MD simulations.

In all ten corresponding ESI complexes, L-idose perfectly maintained its binding conformation, with average RMSD values below 1.5 Å, thus further confirming the reliability of its predicted binding mode. In contrast, GA was not able to maintain its binding disposition in two out of the ten complexes, since a complete detachment of the inhibitor from the ESI complexes was observed. In the eight remaining complexes, the binding disposition of GA was subjected to adjustments of different entities, as indicated by the average RMSD values ranging from 2.5 to 5.8 Å, however the ligand remained anchored to the L-idose-AKR1B1 complex. Of these, complex 2 was found to be the most reliable, showing one of the most stable ligand dispositions (average RMSD = 2.6 Å) and a binding energy (ΔPBSA = −12.8 kcal/mol) at least 3.3 kcal/mol higher than those predicted for the other complexes (Table S3).

**Figure 7:**
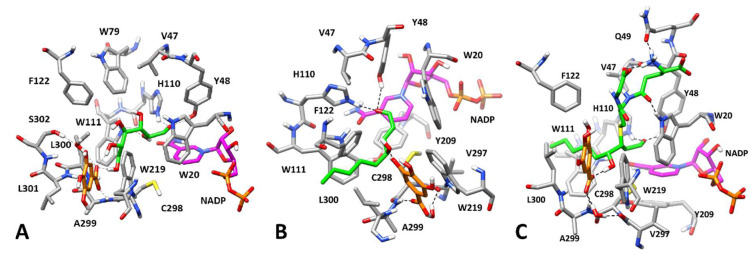
Minimized average structures of AKR1B1 in complexes with GA and different substrates. In each *Panel*, GA is shown in orange, the portion of the cofactor adjacent to the ligand binding site is shown in magenta, and the substrate is shown in green. Relevant protein residues (shown in grey) are indicated. For all components, oxygen atoms are in red, sulfur atoms in yellow, nitrogen atoms in blue, while phosphorous atoms of the cofactor are shown in orange. *Panel* (**A**): AKR1B1 in complex with GA and L-idose; *Panel* (**B**): AKR1B1 in complex with GA and HNE; *Panel* (**C**): AKR1B1 in complex with GA and GSHNE.

As shown in Figure 7A, GA is located in an amphiphilic cavity constituted by W219, C298, A299, L301, S302, and the terminal portion of L-idose. The aromatic ring of the ligand is sandwiched between the side chain of L301 and the indole moiety of W219, forming hydrophobic interactions with the former and a stable π-π stacking with the latter residue. The carboxylic group of GA forms four different H-bonds that anchor the ligand to both the substrate and the protein. The ligand interacts with the two terminal hydroxyl groups of L-idose with two H-bonds that are maintained for almost the entire MD simulation. In addition, a direct H-bond and a water-bridge are formed with the backbone nitrogen of A299 and the hydroxyl group of S302, respectively.

On the basis of its predicted binding mode, we hypothesized that GA may inhibit the reduction of L-idose by blocking the substrate inside the protein binding site, thus hampering the substrate turnover. To test this hypothesis, we used binding energy evaluations to calculate the binding affinity of L-idose in the presence of GA. It is worth noting that the obtained value (−26.4 kcal/mol) was found to be 9 kcal/mol higher than that previously estimated without GA (−17.4 kcal/mol). This result strongly supported our rationale for explaining the inhibitory mechanism of GA on L-idose reduction.

To identify a possible binding mode of GA in the presence of HNE and GSHNE to explain the reduced potency of this molecule in inhibiting the reduction of these substrates by AKR1B1, GA was docked into the previously generated complexes of AKR1B1 bound to HNE and GSHNE (Figure 6A,B, respectively). In the case of the HNE-enzyme complex, 13 different binding modes were obtained for GA, however nine were completely lost during the MD simulation, due to the detachment of GA from the ESI complex, whereas the substrate perfectly maintained its binding mode, with RMSD values below 1.0 Å.

Of the remaining four ESI complexes, complex 4 was found to be the most stable in terms of both ligand RMSD and binding energy calculated for GA (−9.2 kcal/mol), which was significantly higher than that estimated for the other complexes (Table S4). The GA is located between L300 and W219 (Figure 7B) in the same amphiphilic pocket occupied in the presence of L-idose. In fact, in this protein conformation, L301 is replaced by L300 due to its rotation toward the solvent associated with the opening of the specificity pocket. Overall, the binding disposition of GA is similar to the one predicted in the presence of L-idose, and the same hydrophobic/aromatic interactions are observed. However, the carboxylic group of GA is differently oriented and forms a water-mediated interaction with the backbone oxygen of V297, although maintaining the direct H-bond with A299. Despite one of the phenolic groups of the inhibitor forming an additional H-bond with the hydroxyl group of HNE, this interaction did not significantly affect the binding affinity of HNE for the EI complex. In fact, the binding free energy calculated for HNE in the ESI complex (−19.6 kcal/mol) was very similar to the one calculated for the ES complex in the absence of GA (−18.6 kcal/mol).

A very similar result was obtained for the evaluation of GA binding mode in the presence of GSHNE. In this case, eight different binding dispositions were generated by docking, for six of which a complete loss of the inhibitor-protein interactions was observed during MD simulations, while GSHNE always showed an average RMSD below 2.0 Å. The complexes corresponding to the two remaining binding modes showed a different behaviour both from a qualitative and quantitative point of view, and complex 2 was found to be much more reliable in terms of ligand RMSD during the simulation and binding free energy (Table S5).

As shown in Figure 7C, the binding mode predicted for GA in this complex is very similar to that obtained in the presence of HNE. In fact, the inhibitor is again localized between L300 and W219, presenting the same disposition within the amphiphilic pocket and essentially forming the same interactions with both the protein and the substrate. GA maintains the direct H-bond with the backbone of A299, the water-mediated interaction with V297, and the additional H-bond with the hydroxyl group of the HNE moiety of the substrate. The only difference with respect to the ESI complex in the presence of HNE is that the interaction of GA with GSHNE occurs through its carboxylic group. Again, energy assessments of the substrate showed that the binding free energy of GSHNE in the presence of GA (−30.4 kcal/mol) corresponded to the value previously obtained in its absence (−30.3 kcal/mol).

Overall, these results referring to different ESI complexes in the presence of GA, which was predicted to always bind to the same sub-pocket of the AKR1B1 binding site, provided a sound rationale for explaining the differential inhibitory activity of this compound. Therefore, the analysis of the different GA-substrate interactions and the binding energy evaluations performed on the substrates, showed that, while L-idose can form two strong H-bonds with GA, which greatly increase its binding affinity, HNE and GSHNE only form a single and weaker H-bond with the ligand, which does not alter (or only marginally increases) their binding energy. Thus, it is conceivable that GA is able to block L-idose, more than HNE and GSHNE, within the enzyme binding site, thus preferentially inhibiting the reduction of L-idose.

The kinetic analysis showed that EGCG appears to inhibit both L-idose and GSHNE reduction more efficiently than HNE reduction (Figure 1). Moreover, EGCG preferentially binds to the enzyme in the presence of L-idose and HNE, but no preference for the ESI complex was observed when GSHNE is used as a substrate (Table 1). By combining this observation with the higher steric hindrance of EGCG with respect to GA, we hypothesized that EGCG could assume a binding mode able to block L-idose (but not HNE) within the AKR1B1 binding site, similarly to what was predicted for GA, but that the inhibition of GSHNE reduction could be due to the steric hindrance generated by EGCG in its binding disposition, which would prevent the interaction of GSHNE with the enzyme. However, for the two substrates L-idose and HNE whose differential inhibition was characterized, as with GA, by an uncompetitive mode of action (Figure 2 and Figure 3; Table 1), the search for possible dispositions of EGCG at the active site was approached.

In the case of L-idose, the docking of EGCG into the previously obtained AKR1B1-L-idose complex (Figure 6A) generated 46 different clusters of solutions, of which only those showing at least one H-bond interaction between EGCG and L-idose were considered for further studies. Using this filter, 21 different binding modes were studied through MD simulations. A further qualitative filter was then performed, based on the stability of the inhibitor-substrate interactions, in order to identify only the ESI complexes in which EGCG could reliably block L-idose into the catalytic site of the enzyme.

Based on this filter, only seven complexes in which an H-bond between ECGC and L-idose was observed for at least 70% of the MD simulation were retained and subjected to binding energy evaluations. Of these, complex 2, which showed the highest stability in terms of average ligand RMSD during the simulation (2.9 Å), was also found to have the best binding free energy (ΔPBSA = −17.0 kcal/mol) and was thus considered as the most reliable binding mode for EGCG (Table S6).

**Figure 8:**
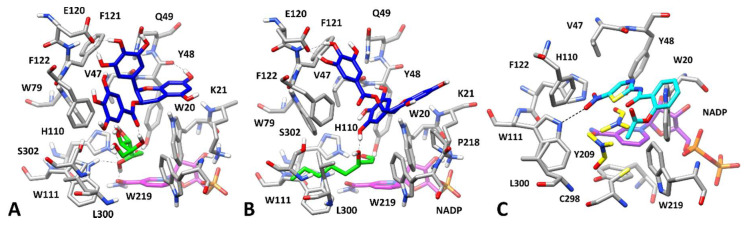
Interactions of AKR1B1 with substrates and/or inhibitors. *Panel* (**A**): Minimized average structures of AKR1B1 in complex with EGCG and L-idose; *Panel* (**B**): Minimized average structures of AKR1B1 in complex with EGCG and HNE; *Panel* (**C**): X-ray structure of AKR1B1 in complex with nitrazoxanide (PDB code 3V35). In each panel, EGCG is shown in blue and the portion of the cofactor adjacent to the ligand binding site is shown in magenta. *Panel* (**A**): L-idose, shown in green; *Panel* (**B**): HNE, shown in green. *Panel* (**C**): Nitazoxanide, shown in cyan. Relevant protein residues (shown in grey) are indicated. For all components, oxygen atoms are in red, sulfur atoms in yellow, nitrogen atoms in blue, while phosphorous atoms of the cofactor are shown in orange.

As shown in Figure 8A, the inhibitor interacts with the portion of the AKR1B1 catalytic site predicted to be occupied by the glutathione moiety of GSHNE, which lies above the substrate and W20. The molecule shows two fundamental H-bonds with E120 that were maintained throughout the MD simulations and enabled the ligand to be anchored to the binding site. In addition, another H-bond with the backbone oxygen of Y48 and a π-π stacking with F122, which helps to stabilize the EGCG disposition, was also observed. Finally, the carbonyl oxygen of the inhibitor formed a stable H-bond with the C5 hydroxyl group of L-idose. However, despite this interaction, the binding free energy associated with the substrate (−17.7 kcal/mol) was found to be comparable to the one estimated in the absence of the inhibitor (−17.4 kcal/mol).

Although this result may limit the reliability of the predicted ESI complex, it is possible that the steric hindrance generated by EGCG, which occupies a major portion of the catalytic site, may contribute to its inhibitory effect by interfering with the exit of L-idose from the binding pocket with a mechanism not easily detectable by simple binding free energy estimations.

With regard to the potential binding of EGCG in the presence of HNE, the docking approach on the previously generated AKR1B1-HNE complex (Figure 6B), produced 40 different clusters of poses for EGCG. In the attempt of pre-filtering the 40 obtained docking solutions, we searched for binding modes similar to the one assumed in the ESI complex with L-idose, since we believed that even EGCG would assume a comparable binding mode both in the presence of L-idose and HNE, as predicted for GA. None of the generated binding dispositions closely resembled that shown in Figure 8A. However, 14 binding poses in which EGCG was located in the same portion of the binding site were identified and studied through MD simulations.

Unfortunately, the analysis of the MD results in terms of the RMSD of ligand disposition during the simulation and the binding free energy evaluations did not suggest for EGCG a binding mode clearly more reliable than others (Table S7). However, we were able to identify complex 14 as a promising model of the AKR1B1-HNE-EGCG interaction, which is consistent with the previously obtained results and with the mechanism of action of the differential inhibitor. In fact, complex 14 showed the best binding free energy value (−16.7 kcal/mol) and one of the highest stabilities in terms of average RMSD of ligand disposition during MD. Moreover, the binding disposition of EGCG in this complex was found to be similar to that observed in the presence of L-idose (compare Figure 8A,B). In particular, the coumarin moiety of the inhibitor was located above W20 and was again anchored to the protein thanks to a double H-bond with the carboxylic group of E120. The ligand also maintained the π-π stacking with F122 and an additional H-bond with the backbone oxygen of V47, which stabilized its disposition.

Finally, EGCG formed a further H-bond with the hydroxyl group of HNE, which maintained an extremely similar binding mode to those predicted in the presence of GA and in the absence of any inhibitor (Figure 7B and Figure 6B, respectively). Despite this inhibitor-substrate interaction, the substrate-protein binding energy calculated for HNE in the ESI complex (−18.8 kcal/mol) was found to be almost unchanged with respect to the one estimated in the absence of EGCG (−18.6 kcal/mol). Moreover, as shown in Figure 8B, EGCG seems to determine a significantly lower steric occlusion of the binding site with respect to the ESI complex with L-idose, which should facilitate the exit of HNE from the catalytic pocket.

Nevertheless, from the analysis of the structures of the ternary complexes of EGCG with L-idose and HNE (Figure 8), EGCG would seem to exert a significant steric hindrance at the level of the region in which the glutathionyl moiety of GSHNE is allocated (Figure 6C). Taken together, these results would seem to concur with the differential inhibitory activity of ECGC. This compound could inhibit the reduction of GSHNE and L-idose catalyzed by AKR1B1 mainly by steric hindrance, preventing the binding of GSHNE to the enzyme, and, also through the formation of H-bonds, blocking L-idose in the catalytic pocket. On the other hand, EGCG could not be able to prevent the dissociation of HNE from the enzyme and thus to inhibit the catalytic activity of AKR1B1 for this type of substrate.

These results are also consistent with the binding mode into the AKR1B1 catalytic site of the known ligand nitazoxanide (PDB code 3V35), which was demonstrated to bind to the ESI complex in the presence of GAL. This compound could not be co-crystallized with AKR1B1 in the presence of the substrate; however, an X-ray complex obtained in the presence of dimethylformamide (Figure 8C) showed a binding mode in which the inhibitor is localized above the anion binding pocket, in a similar portion of the catalytic site to the one occupied by EGCG in our predicted ESI complexes.

As a final step in our computational analysis, we studied the inhibitory mechanism of EGC, which acts as a classic inhibitor affecting the AKR1B1-mediated reduction of L-idose, HNE, and GSHNE with comparable efficiency. The kinetic analysis revealed that EGC does not show a marked preferential binding for the ES complexes, and actually behaves as a competitive inhibitor in the case of GSHNE. Thus, EGC was docked into the X-ray structure of AKR1B1 in the absence of substrates.

In this case, it was not hard to identify a reliable binding mode for the inhibitor. In fact, only five different binding dispositions were generated by docking and were then analyzed through MD simulations. One of these (complex 3) was significantly more reliable that the others in terms of ligand RMSD, with an average value of 0.7 Å, and showed a binding free energy value of −18.0 kcal/mol that outperformed by at least 4.0 kcal/mol those of complexes 1, 2, and 4 (Table S8). Complex 5 presented a rather similar binding energy (−16.7 kcal/mol) only because during the MD simulation the ligand rapidly converged into the binding mode assumed in complex 3, thus further confirming the reliability of this latter complex.

**Figure 9:**
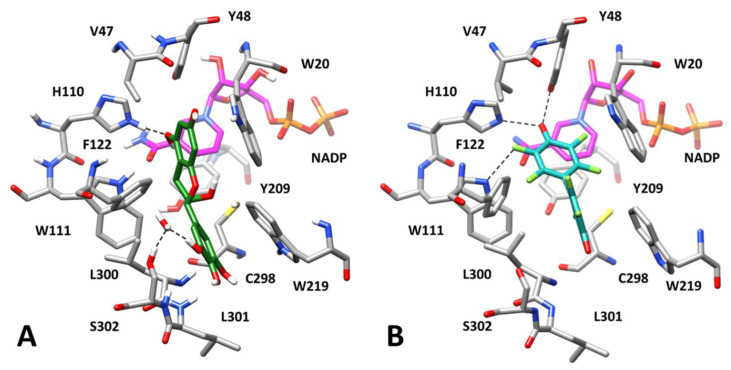
Interactions of AKR1B1 with inhibitors. *Panel* (**A**): Minimized average structures of AKR1B1 in complex with EGC, shown in green. *Panel* (**B**): X-ray structure of AKR1B1 in complex with JF0064, shown in cyan (PDB code 4IGS). In each panel, the portion of the cofactor adjacent to the ligand binding site is shown in magenta. Relevant protein residues (shown in grey) are indicated. For all components, oxygen atoms are in red, sulfur atoms in yellow, nitrogen atoms in blue, while phosphorous atoms of the cofactor are shown in orange.

As shown in Figure 9, the bicyclic core of ECG interacts with the anion binding pocket of the enzyme, while placing the phenolic ring in the amphiphilic pocket which is likely the binding site of GA in the presence of L-idose, HNE, and GSHNE. The main interactions formed by EGC are represented by a direct H-bond with H110, a water mediated H-bond with the hydroxyl group of S302 and π-π stacking interactions with both W20 and W219. Based on the predicted binding mode, ECG likely blocks the access to the catalytic site of AKR1B1, and in particular to the catalytic residues of the anion binding pocket. Therefore, this prevents the enzymatic reduction of all AKR1B1 substrates, regardless of their structure and binding conformations. This result is consistent with the classic inhibitory character of ECG. In this regard, it is worth noting the consistency of the predicted binding mode of ECG with the experimental binding disposition of the known inhibitor JF0064, which presents a structural similarity with EGC and was co-crystallized with AKR1B1 (PDB code 4IGS). This AKR1B1 inhibitor is very active at sub micromolar levels, since it is endowed with a deprotonated phenolic group, which can form charged H-bonds with both Y48 and H110, a further H-bond with W111 through one of its fluorine atoms, and additional water-mediated interactions. The reduced interactions of ligand-protein interactions shown for EGC with respect to JF0064 could justify the lower potency of the natural compound as an AKR1B1 inhibitor.

Overall, our computational study led to the identification of reliable binding modes to AKR1B1 for D-glucose and the three representative substrates used in our experimental assays: L-idose, HNE, and GSHNE. Our results also provide an explanation of the mechanism of action of the differential inhibitors GA and EGCG, in contrast to EGC, as highlighted by the classical kinetic model used in our study.

### 3.4. Differential Inhibition of AKR1B1 in Green Tea Extracts

**Figure 10:**
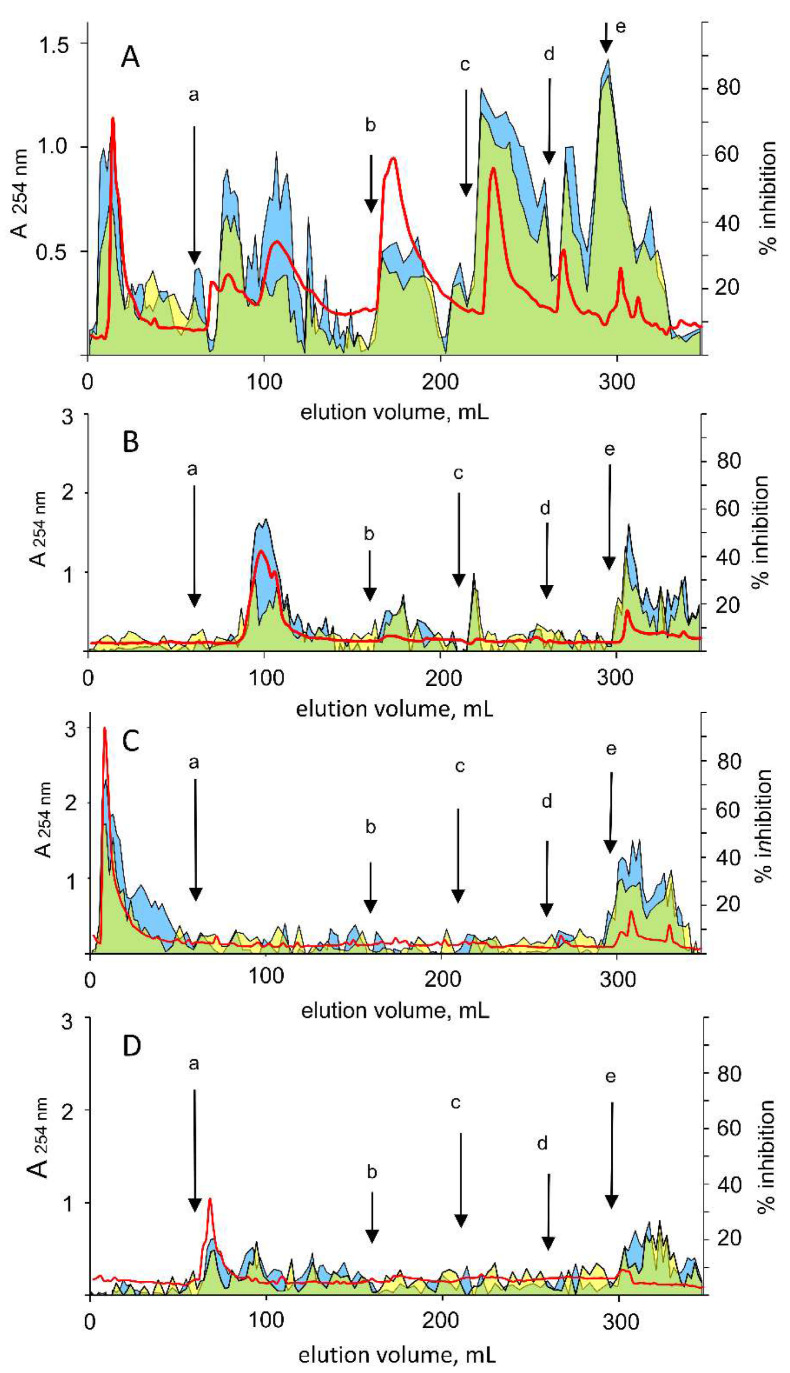
Chromatographic fractionation of green tea extract and AKR1B1 differential inhibition. *Panel* (**A**): A water-soluble fraction of a green tea extract (see Materials and Methods) was loaded onto a Bondelut C18 column. The elution was performed at the indicated points by a series of isocratic steps of % methanol in water (v:v) as follows: *a*, 10; *b*, 20; *c*, 30; *d*, 50; and *e*, 100 (see Methods for details). The separation profile monitored at 254 nm (red line) and the % inhibition exerted by individual fractions measured using L-idose (blue area) and HNE (yellow area) as substrates, are reported. The green area refers to the overlap of blue and yellow areas. The percentage inhibition refers to the activity measured in the presence of the eluted fraction with respect to the activity measured in the absence of the fraction. *Panels* (**B**–**D**) refer to the chromatographic analysis performed in the conditions described in *Panel* (**A**) of commercial standards of EGCG, GA, and EGC, respectively. The standards were dissolved (10 mM) in water and loaded (5 µmoles) on the column.

As EGCG is the most represented catechin in the green tea, the tea extract was examined for a differential inhibitory action. The water-soluble fraction of a green tea extract (see Materials and Methods) was fractionated through hydrophobic chromatography by a stepwise isocratic elution. A typical chromatogram is reported in Figure 10A. This shows an articulated elution profile with fractions able to inhibit AKR1B1 and including a number of fractions that exert differential inhibition between L-idose and HNE reduction.

Taking into account the elution profiles obtained for commercial EGCG, GA, and EGC subjected to chromatographic analysis in the same conditions as above (Figure 10, *Panels B*, *C*, and *D*, respectively), fractions of the extract showing differential activity, with elution volumes ranging between 90 and 120 mL and between 5 and 30 mL were associated, although not unequivocally, with EGCG and GA, respectively. In fact, an increase in the differential inhibitory peak at elution volumes of 90–120 mL was observed when the extract was supplemented with 5 µmoles of commercial EGCG (data not shown). In addition to EGCG and GA, other green tea components, eluted in the volume ranges 170–190 mL and 230–260 mL, appeared to exert a differential inhibitory action. This occurred despite the presence of classical ARIs, which likely contribute to an underestimation of the differential effectiveness of the ARDIs present. Clarification of the composition in ARDIs of the tea extracts, as well as the potential structural and functional interactions between components, leading to the emergence of properties of the sample different from the algebraic sum of the properties of its single components, when separately investigated, is not an easy task and certainly merits further research.

## 4. Conclusions

Our results reveal new features of EGCG in differentially inhibiting aldose reductase. Massive aldohexoses reduction, as can occur in hyperglycaemic conditions, and the proinflammatory event associated with the reduction of GSHNE to GSDHN can thus be inhibited at a greater extent than the HNE reduction, preserving the detoxifying role of the enzyme. The GA moiety of EGCG, which also intervenes in polyols generation while preserving the detoxification ability of AKR1B1 in reducing toxic aldehydes, appears to be less effective in preventing GSDHN generation. The different disposition of the various substrates, as shown by computational investigations, is the basic evidence that suggests that AKR1B1 is susceptible to an intra-site differential inhibition. Finally, we believe that our results provide new insights into the structural requirements of molecules to exploit the ARDI activity, and also highlight that green tea has additional functional features that may explain its beneficial action against hyperglycemic-linked cell damage as well as inflammation phenomena.

## Figures and Tables

**Table 1:** Inhibition constants for EGCG, GA, and EGC for the inhibition of **l**-idose, HNE, and GSHNE reduction.

		ECGC			GA			EGC	
Substrate	K_i_’	K_i_	K_i_/K_i_’	K_i_’	K_i_	K_i_/K_i_’	K_i_’	K_i_	K_i_/K_i_’
L-idose	61 ± 9	425 ± 64	7.0	103 ± 7	n.d.	n.a.	496 ± 35	1239 ± 2 33	2.5
HNE	116 ± 11	n.d.	n.a.	253 ± 23	n.d.	n.a.	686 ± 63	1682 ± 289	2.5
GSHNE	144 ± 22	196 ± 17	1.3	793 ± 102	n.d.	n.a.	n.d.	553 ± 87	n.a.

The values of the constants, expressed in µM, derived from the steady state kinetic analysis of Figure 2, Figure 3, and Figure 4, are reported as the mean ± SD; n.d.: Not detectable; n.a.: Not applicable. *K_i_* and *K’_i_* stand for *^app^K_i_* and *^app^K’_i_*, respectively, as defined in Section 2.6.

## Supplementary Materials

The following are available online at https://www.mdpi.com/2218-273X/10/7/1003/s1. Table S1: MM-PBSA results for AKR1B1-3S,4R-GSHNE complexes; Table S2: MM-PBSA results for AKR1B1-3R,4R-GSHNE complexes; Table S3: MM-PBSA results for AKR1B1-GA-IDOSE complexes; Table S4: MM-PBSA results for AKR1B1-GA-HNE complexes; Table S5: MM-PBSA results for AKR1B1-GA-GSHNE complexes; Table S6: MM-PBSA results for AKR1B1-EGCG-IDOSE complexes; Table S7: MM-PBSA results for AKR1B1-EGCG-HNE complexes; Table S8: MM-PBSA results for AKR1B1-EGC complexes.
