# Supplementary material for: Aldose Reductase Differential Inhibitors in Green Tea
Source: Biomolecules. 2020 Jul 6;10(7):1003. doi: 10.3390/biom10071003 (PMC7407822; doi:10.3390/biom10071003)
Supplement: Supplementary file 1 [file biomolecules-10-01003-s001.pdf]

biomolecules

MDPI

Aldose reductase differential inhibitors in green tea

Supplementary Materials

Table S1. MM-PBSA results for the ten different AKR1B1-3S,4R-GSHNE. The average RMSD of GSHNE disposition during the simulation is also reported

**Complex**	**ΔPBSA (kcal/mol)**	**Ligand RMSD (Å)**
**9**	-26.6	2.8
**6**	-25.6	2.4
**2**	-24.7	2.2
**8**	-24.0	4.1
**5**	-22.6	2.6
**3**	-21.5	3.2
**7**	-21	3.1
**4**	-20.6	3.8
**10**	-19.1	3.4
**1**	-16.1	4.3

Table S2. MM-PBSA results for the twelve different AKR1B1-3R,4R-GSHNE. The average RMSD of GSHNE disposition during the simulation is also reported

**Complex**	**ΔPBSA (kcal/mol)**	**Ligand RMSD (Å)**
**3**	-30.0	1.9
**1**	-27.0	2.6
**6**	-26.9	2.4
**2**	-25.5	3.6
**8**	-24.3	2.6
**10**	-23.8	4.3
**9**	-20.2	3.0
**11**	-19.9	2.2
**12**	-19.4	3.7
**4**	-19.1	3.9
**7**	-18.7	3.1
**5**	-16.3	2.8

Table S3. MM-PBSA results for the eight different AKR1B1-GA-Idose complexes. The average RMSD of GA disposition during the simulation is also reported.

Complex[a]	ΔPBSA (kcal/mol)	Ligand RMSD (Å)
**2**	-12.8	2.6
**6**	-9.5	2.5
**8**	-9.0	4.5
**3**	-7.0	5.8
**4**	-5.2	5.4
**5**	-4.9	4.0
**1**	-4.1	3.3
**7**	-3.6	3.0

[a]Only stable complexed are considered

Table S4. MM-PBSA results for the four different AKR1B1-GA-HNE complexes. The average RMSD of GA disposition during the simulation is also reported.

Complex[a]	ΔPBSA (kcal/mol)	Ligand RMSD (Å)
**4**	-9.2	2.9
**3**	-2.5	7.0
**1**	-2.0	6.2
**2**	-1.9	4.7

[a]Only stable complexed are considered

Table S5. MM-PBSA results for the two different AKR1B1-GA-GSHNE complexes. The average RMSD of GA disposition during the simulation is also reported.

Complex[a]	ΔPBSA (kcal/mol)	Ligand RMSD (Å)
**2**	-12.8	3.4
**1**	-1.8	7.7

[a]Only stable complexed are considered

Table S6. MM-PBSA results for the seven different AKR1B1-EGCG-L-idose complexes. The average RMSD of EGCG disposition during the simulation is also reported.

Complex	ΔPBSA (kcal/mol)	Ligand RMSD (Å)
**2**	-17.0	2.9
**3**	-12.5	5.8
**6**	-12.1	5.3
**1**	-11.7	5.0
**5**	-11.5	4.5
**7**	-11.3	3.6
**4**	-8.8	3.8

Table S7. MM-PBSA results for the fourteen different AKR1B1-EGCG-HNE complexes. The average RMSD of EGCG disposition during the simulation is also reported.

Complex[a]	ΔPBSA (kcal/mol)	Ligand RMSD (Å)
**14**	-16.7	3.5
**3**	-16.0	4.6
**4**	-15.6	3.6
**6**	-15.1	1.7
**9**	-15.1	3.4
**11**	-14.9	5.4
**1**	-13.4	3.6
**2**	-11.5	6.0
**5**	-10.9	5.1
**7**	-10.8	4.1
**12**	-10.7	4.0
**10**	-10.1	8.0
**13**	-9.5	6.8
**8**	-8.7	3.4

Table S8. MM-PBSA results for the five different AKR1B1-EGC complexes. The average RMSD of EGC disposition during the simulation is also reported.

Complex	ΔPBSA (kcal/mol)	Ligand RMSD (Å)
**3**	-18.0	0.7
**5**[a]	-16.7	2.0
**1**	-14.0	1.8
**4**	-7.2	3.9
**2**	-6.6	2.9

[a]converged into complex 3
